# The influence of chemical structure on thermal properties and surface morphology of polyurethane materials

**DOI:** 10.1007/s11696-017-0358-6

**Published:** 2017-12-08

**Authors:** Joanna Brzeska, Magda Morawska, Aleksandra Heimowska, Wanda Sikorska, Wojciech Wałach, Anna Hercog, Marek Kowalczuk, Maria Rutkowska

**Affiliations:** 1 Department of Commodity Industrial Science and Chemistry, Gdynia Maritime University, 83 Morska Street, 81-225 Gdynia, Poland; 2 Centre of Polymer and Carbon Materials, Polish Academy of Sciences, 34 M. Curie-Sklodowska Street, 41-819 Zabrze, Poland; 3 Faculty of Science and Engineering, School of Biology, Chemistry and Forensic Science, University of Wolverhampton, Wolverhampton, WV1 1SB UK

**Keywords:** Polyurethane structure, Polymer blends, Thermal properties, Surface morphology

## Abstract

The surface morphology and thermal properties of polyurethanes can be correlated to their chemical composition. The hydrophilicity, surface morphology, and thermal properties of polyurethanes (differed in soft segments and in linear/cross-linked structure) were investigated. The influence of poly([*R*,*S*]-3-hydroxybutyrate) presence in soft segments and blending of polyurethane with polylactide on surface topography were also estimated. The linear polyurethanes (partially crystalline) had the granular surface, whereas the surface of cross-linked polyurethanes (almost amorphous) was smooth. Round aggregates of polylactide un-uniformly distributed in matrix of polyurethane were clearly visible. It was concluded that some modification of soft segment (by mixing of poly([*R*,*S*]-3-hydroxybutyrate) with different polydiols and polytriol) and blending of polyurethanes with small amount of polylactide influence on crystallinity and surface topography of obtained polyurethanes.

## Introduction

The surface morphology of polymeric material is an important factor which influences on many properties of object made of polymer. Hydrophilicity, roughness, presence of functional groups and crystallinity of polymer affected its biocompatibility in medicine application, on degradability in systems of fertilizer deliver for agriculture, and so on. The interaction between polymer and surrounded environment is strictly connected with the surface structure and topography. At the first-stage environment, factors act on polymer surface. Therefore, hydrophobic surface with appropriate roughness on one hand facilitates the attachment of albumin on the artificial implant (Wang et al. 2012), and, on the other hand, increases the area of action of degradative enzymes. The hydrophilicity of polymer is favourable for its degradation in water conditions. Therefore, the material for constructed object is chosen after taking under consideration all properties required for the special use.

Polyurethane (PUR) is a good material that can be easy synthesized according to application requirements. The substrates for its synthesis are chosen depending on needs and they affect physical, chemical, and biological properties of polyurethanes (PURs). All these properties are strictly connected to each other. For example, introducing of fluorocarbon chains into PURs leads to obtaining of the hydrophobic material with increased contact angle, and with good chemical resistance (Wang et al. 2016; Licchelli et al. 2011). Whereas PURs with poly(dimethylsiloxane), which is used as soft segments, are compatible with blood, with good resistance to fibrinogen adsorption, and favour albumin adsorption. Different types of used poly(dimethylsiloxane) influence on obtained contact angle and surface topography (Pergal et al. 2014a).

Obviously, despite of kind of substrates used for PUR synthesis, also the soft and hard segments ratio, mobility of chains, and procedure/conditions of preparing influenced on their properties. For example, Pergal and others (Pergal et al. 2014a) concluded that contact angle decreased with increasing the content of hard segments in PUR bulk.

It is clear that substrates influence on the type of formed nanostructures, such as: nanodomains in amorphous PURs and crystalline structures, which next affects the properties of polymer (Janik 2010). The first platform of environment–polymer interactions takes place on macro and microscale. It is interesting how the presence of nanostructures in PUR bulk acts on the morphology of micro and macrosurface of the samples, moulded under the same conditions and with the same thickness.

In the paper, the results of thermal analyses (using differential scanning calorimetry) and surface characterization (done by scanning electron microscope, optical microscope, and goniometer instrument) for seven types of PUR material were presented. Samples were differed in linear/cross-linked structure and in composition of soft segments (build with polyester or polyester–ether). In addition, the influence of physical blending of PURs with poly([d,l]-lactide) on their properties was estimated.

### Experimental

## Materials

**Fig. 1:**
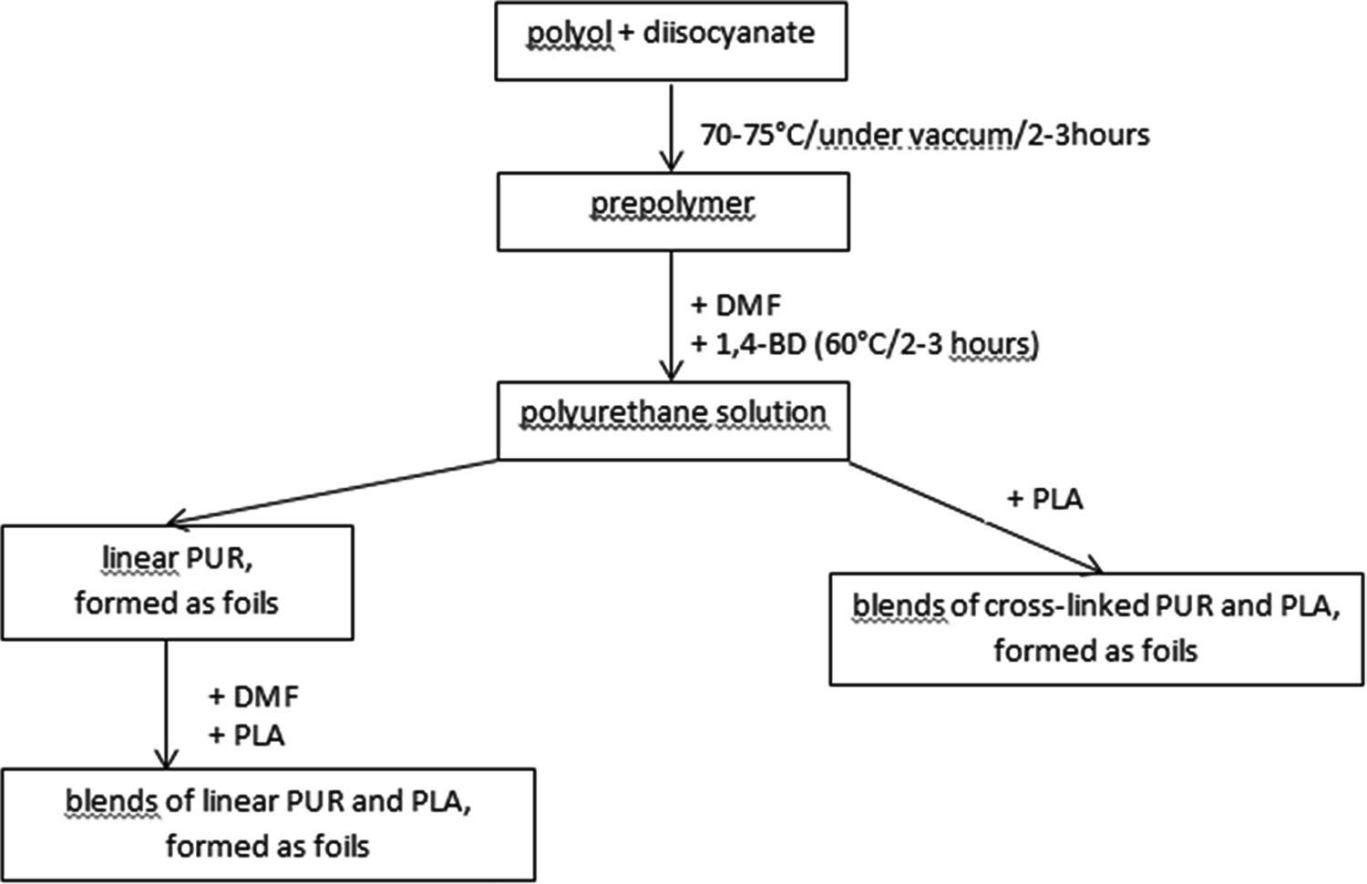
Simplified scheme for obtaining of blends of linear and cross-linked PURs with polylactide

PURs were synthesized in the two-step polyaddition reaction and next blended with polylactide, according to scheme below (Fig. 1).

Soft segments of PURs were built with polyols: (i) polycaprolactone diol (PCL_diol_) (*M*_n_ 1900, Aldrich) and synthetic poly([*R*,*S*]-3-hydroxybutyrate) (*R*,*S*-PHB) (*M*_n_ 1700,) or polyoxytetramethylene diol (PTMG) (*M*_n_ 2000, Aldrich) and *R*,*S*-PHB in case of linear PURs, or (ii) polycaprolactone triol (PCL_triol_) (M_n_ 900, Aldrich) and *R*,*S*-PHB for cross-linked PURs. Synthetic *R*,*S*-PHB was obtained previously by anionic ring opening polymerization of (*R*,*S*)-ß-butyrolactone initiated by 3-hydroxybutyric acid sodium salt/18-crown-6 complex at room temperature and terminated with 2-bromoethanol (Arslan et al. 1999). Hard segments of all PURs were synthesized with aliphatic 4,4′-methylene dicyclohexyl diisocyanate (H_12_MDI) (Aldrich) and 1,4-butanediol (1,4-BD) (Aldrich).

At first step, mixture of polyols was reacted with diisocyanate under vacuum (Fig. 1). Next, obtained prepolymer was dissolved in *N*,*N*-dimethylformamide (DMF) and appropriate amount of chain extender was added (for maintaining final molar ratio of NCO:OH = 1:1). PURs were formed into foils on Teflon plates in the vacuum drier, after evaporation of DMF.

Blends of linear PUR with poly([d,l]-lactide) (PLA) (*M*_w_ 18,000–28,000, Aldrich) were obtained by mixing both (dissolved in DMF) polymers. In case of cross-linked PURs, PLA was introduced into PUR solution (in DMF) at the end of second step of synthesizes.

All samples, formed into foils with thickness 0.4–2.0 mm, were seasoned at least 2 weeks before the investigations.

### Methods

#### Differential scanning calorimetry (DSC)

Thermal properties of PURs and their blends were determined using the Setaram thermal analyzer. Indium and lead were used for calibration. The specimens (with mass about 20 mg) were sealed in aluminum pans and scanned from 20 to 200 °C with the heating rate of 10 °C/min. All experiments were made in a flow of dry N_2_.

#### Contact angle

Surface wettability of polymers was estimated using the method of sessile drop with a droplet size of about 15 μL. The drop was immersed on surface, formed in the same conditions (samples with the same thickness, formed at the same temperature and time on Teflon plates, and with the same rate of solvent evaporation), between polymer and air (surface at the film–air interface). Tests were performed using a goniometer Cam 101 KSV Instruments and were carried out under air and at room temperature.

#### Microscopic observation

The polymer surfaces were observed using high-resolution environmental scanning electron microscope (SEM) and optical microscope (OM). SEM studies were performed using of a Quanta 250 FEG (FEI) high-resolution environmental scanning electron microscope operated at 10 kV acceleration voltages. The samples were observed under low vacuum (80 Pa) using a secondary electron detector (Large Field detector) and backscattered electron (BSE) detector. Energy-dispersive spectra (EDS) were collected by Apollo SD detector and Genesis V6.20 software (EDAX). Mass concentrations were calculated for carbon and oxygen from five different places for each sample. The polymers surface was also observed in reflected light with an optical microscope Nikon Alphaphot-2YS2 connected with digital photo camera Casio QY2900UX, at magnification 1:300.

## Results and discussion

Linear and cross-linked PURs, with the same hard segments, were synthesized and blended with poly([d,l]-lactide), as it is schematically presented in Fig. 1. PURs were named according to linear (l) and cross-linked (c) structure and to the soft segment building. The nomenclature and composition of PURs and blends are presented in Table 1.

**Table 1:** Name and composition of PURs and their blends with PLA

Sample	Polyols	Substrates used for HS building	Molar ratio of NCO:OH in prepolym.	Amount of PCL_triol_ in PUR (wt%)	Amount of PLA in blend (wt%)
l-PUR_HBCL_	20 wt% *R*,*S*-PHB + 80 wt% PCL_diol_	H_12_MDI +1,4-BD	2:1	0	0
l-PUR_HBCL_/PLA					5
l-PUR_HBTMG_	20 wt% *R*,*S*-PHB + 80 wt% PTMG		2:1	0	0
l-PUR_HBTMG_/PLA					5
c-PUR_CL_	100 wt% PCL_triol_		4:1	33	0
c-PUR_HBCL_	10 wt% *R*,*S*-PHB + 90 wt% PCL_triol_		4:1	30	0
c-PUR_HBCL_/PLA					5

*HS* hard segment, *R,S-PHB* poly([*R*,*S*]-3-hydroxybutyrate), *PCL*_*dio*l_ polcaprolactone diol, *PCL*_*triol*_ polycaprolactone triol, *PTMG* polyoxytetramethylene diol, *PLA* poly([d,l]-lactide), *H*_*12*_ *MDI* 4,4′-methylene dicyclohexyl diisocyanate, *1,4-BD* 1,4-butanediol

**Fig. 2:**
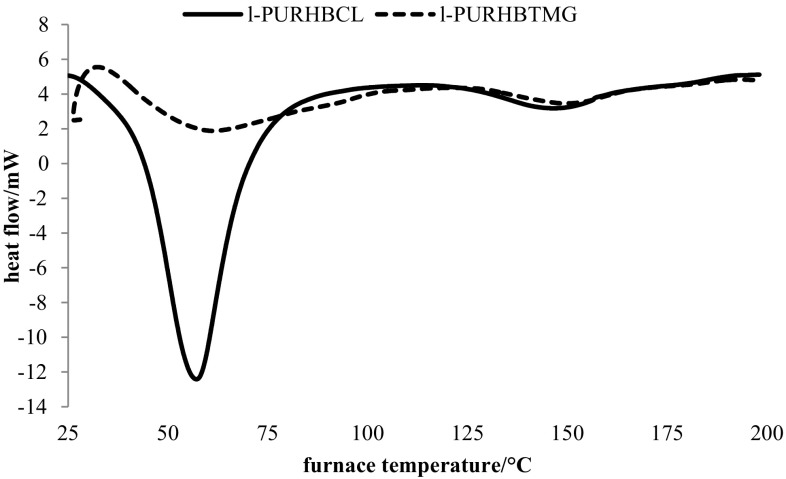
Exemplar DSC thermograms of linear PURs

Results of contact angle and thermal properties of linear and cross-linked PUR materials are presented in Table 2 and Fig. 2.

**Table 2:** Contact angle and thermal properties of PURs and their blends with polylactide

Sample	Contact angle (°) ( ± SD)	*T*_m1_^**^ (°C)	ΔH_1_^**^ (J/g)	*T*_m2_^**^ (°C)	ΔH_2_^**^(J/g)
l-PUR_HBCL_	–*	58.6	48.9	146.3	5.6
l-PUR_HBCL_/PLA	79.8 ± 1.6	48.3	26.8	120.5	0.2
l-PUR_HBTMG_	76.7 ± 5.9	60.4	18.5	149.7	3.7
l-PUR_HBTMG_/PLA	83.0 ± 1.3	A broad peak with max. at 62.0 and 84.0	12.8	120.1 129.4	0.3 0.3
c-PUR_CL_	92.7 ± 4.1	59.5	7.5	107.6	6.6
c-PUR_HBCL_	73.3 ± 4.1	54.2	9.1	88.1 121.4	1.8 0.6
c-PUR_HBCL_/PLA	77.3 ± 4.8	51.9	18.6	121.9	0.01

*Due to the high surface roughness, the results of the determination of the contact angle were unrepeatable

**Means melting temperature and enthalpy of soft (*T*_m1_, *ΔH*_1_) and hard (*T*_m2_, *ΔH*_2_) segments

Two endothermic transitions are observed on DSC thermograms of PURs and their blends: first connected with melting of soft segments (*T*_m1_) and second—with melting of hard segments (*T*_m2_).

It is well known that the composition of soft segments of PURs strongly affects their thermal properties. Crystallinity of polyurethane based on semi-crystalline oligomeric PCL_diol_ (l-PUR_HBCL_) was much higher compared to l-PUR_HBTMG_ based on PTMG_diol_ (Table 2 and Fig. 2). The melting enthalpy of soft segments (ΔH_1_) of l-PUR_HBTMG_ was significantly lower than ΔH_1_ of l-PUR_HBCL_.

Blending of linear PURs with the small amount (only 5 wt%) of amorphous polylactide reduced their crystallinity—melting enthalpy of soft (ΔH_1_) and hard (ΔH_2_) segments decreased (Table 2). Polylactide chains disorganized the ordering of oligomerols, building soft segments, and in consequence the crystalline structures could not be formed. Moreover, melting temperature of hard segments moved to the lower value.

Different situation was observed for cross-linked PURs. Cross-linked c-PUR_CL_, based only on PCL_triol_ in soft segments, was characterized by low crystallinity of soft segments (ΔH_1_ 7.5 J/g). The low endopeak (ΔH_1_ 6.6 J/g) was observed at high temperature (107.6 °C) on thermograms. It was probably related to the ordering of the chains in the hard segment domains (amount of hard segments in cross-linked PURs was higher than in linear PURs). Chains in cross-linked polymers were stiffened by chemical cross-links (in soft segments) and by hydrogen bonds (in hard segments) and could not order, and the crystallites in soft segments were not formed. Introducing of *R*,*S*-PHB into soft segments increased the distance between nodes of cross-links and increased the mobility of chains. In consequence, they could arrange and form the crystallites, what was observed as a small increase of melting enthalpy of soft segments (to 9.1 J/g). In addition, the polylactide chains, mixed with PUR network, acted as plasticizers and facilitated the chains ordering, what was observed in the increasing of crystallinity c-PUR_HBCL_/PLA (Table 2).

Contact angle of water was used as a criterion for the evaluation of hydrophilicity/hydrophobicity of polymer surface. The surface of samples of linear l-PUR_HBCL_ was too rough for reliable estimation of contact angle. Because of this, the influence of blending of l-PUR_HBCL_ with amorphous PLA on contact angle could not be estimated.

The foils were formed after evaporation of DMF from polyurethane solution poured previously on Teflon plates. As it was said before contact angle was measured by immersion of water drop on surface, formed under the same conditions and between polymer and air. It was also showed that the soft segments of l-PUR_HBCL_ had the highest tendency for crystallization (Table 2), and simultaneously, irregularities of its surface were very high. It was suggested that the roughness was strongly affected by the formation of crystalline structures in PUR bulk.

Comparison of contact angle of blends l-PUR_HBCL_/PLA and l-PUR_HBTMG_/PLA (Table 2) confirmed that the crystallinity was only one of the factors affecting the surface hydrophilicity. The surface of l-PUR_HBTMG_/PLA was more hydrophobic (with higher contact angle) despite the crystallinity of this blend was lower than l-PUR_HBCL_/PLA. This was because of the roughness of the surface, which influenced on polymeric properties, such as the albumin adsorption in medical application. Sirkecioglu et al. (2014) stated that roughness played even more important role than the hydrophilicity.

Blend of cross-linked PUR (based on PCL and *R*,*S*-PHB) with PLA was a bit more hydrophilic than linear l-PUR_HBCL_/PLA (Table 2).

**Fig. 3:**
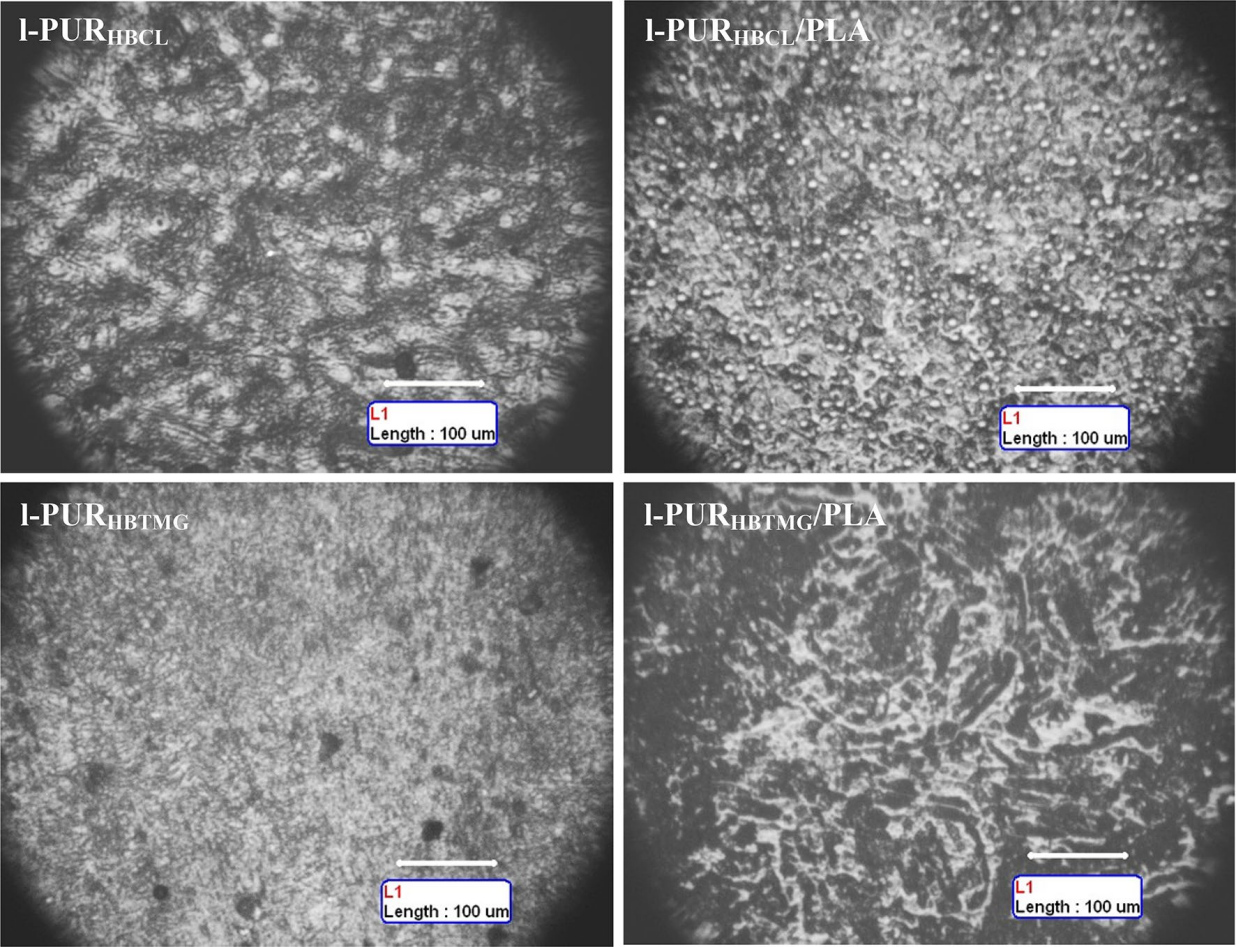
OM micrographs of linear PURs and their blends

**Fig. 4:**
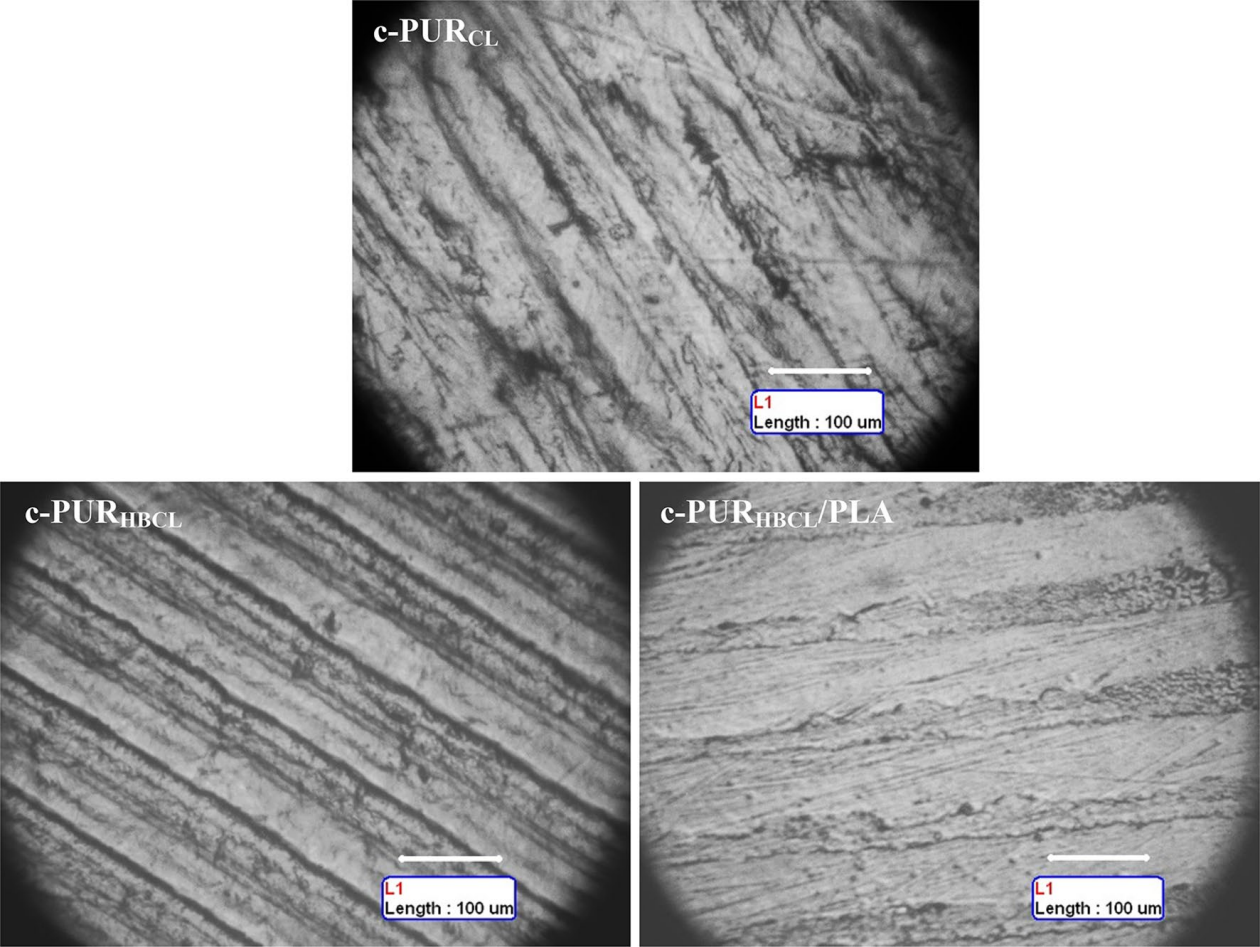
OM micrographs of cross-linked PURs and blend with PLA

The topography of surface of linear and cross-linked PURs and their blends were completely different. The linear PURs had the granular surface, whereas surface of cross-linked PURs was smooth with the regular oriented stripes looking like depressions (Figs. 3, 4).

The surface of samples of linear PURs was rough with varying degrees of roughness. PUR based on PCL (l-PUR_HBCL_) had a rock-like surface, which was much more granular than l-PUR_HBTMG_. As it was said before, PURs based on oligomeric PCL diol had the tendency to crystallization (Table 2). The presence of crystallites in PUR bulk caused that the surface formed at the film–air interface could not be created as smooth. Sharp and narrow peak in DSC thermogram and high melting enthalpy of soft segments indicated that the crystallites were large and uniform (Fig. 2). This resulted in bulges on the surface of the PUR after solvent evaporation. The endotherm of soft segments melting on DSC thermogram of l-PUR_HBTMG_ was wider and much smaller than for l-PUR_HBCL_. Thus, the crystallites in bulk were smaller and more varied, what caused that the surface of the l-PUR_HBTMG_ was smoother than l-PUR_HBCL_ (Fig. 3).

Also blending of l-PUR_HBCL_ with PLA caused that surface was less rough, what was in agreement with the results of the study DSC (Table 2). The melting enthalpy of l-PUR_HBCL_/PLA was almost twice lower than l-PUR_HBCL_. However, the surface of the l-PUR_HBTMG_/PLA was more rugged and irregular than l-PUR_HBTMG_, despite of its lower crystallinity. The presence of PLA chains in PUR network decreased ordering of soft and hard segments. In consequence, it was supposed that only small part of soft domains could form the crystallite structures. However, despite they were small and with variable sizes (what was showed on DSC thermograms), it caused that surface, observed in micro scale, was so irregular. Moreover, the roughness of surface could be also the result of the chemical bonding of incompatible polymers, which next self-assemble into micro- and nanostructures (Joki-Korpela and Pakkanen 2011). For example, incompatibility of PLA and PTMG, and the presence of two associated with them glass transition temperatures, allowed to obtain the copolymer having characteristics of self-assembly (Yan et al. 2013).

The surface of cross-linked PURs looked completely different. It was homogeneous and flat with oriented and parallel strips looked like depression (Fig. 4). The visible strips were the result of mapping the surface of a Teflon plate to which a solution of PUR was poured while forming a film. Even the samples were quite thick (0.4–2.0 mm) and micrographs were taken at the film–air interface in reflected light, the transparency of them caused that visible mapping. Taking under consideration the places between stripes, it was clear that the introduction of *R*,*S*-PHB into the structure of the soft segments and blending of PUR with PLA increased the uniformity and smoothness of the sample. Surface of c-PUR_CL_ was the most irregular, rough, and uneven among all cross-linked PURs, what was the reason of high contact angle (92.7°) (Table 2).

**Fig. 5:**
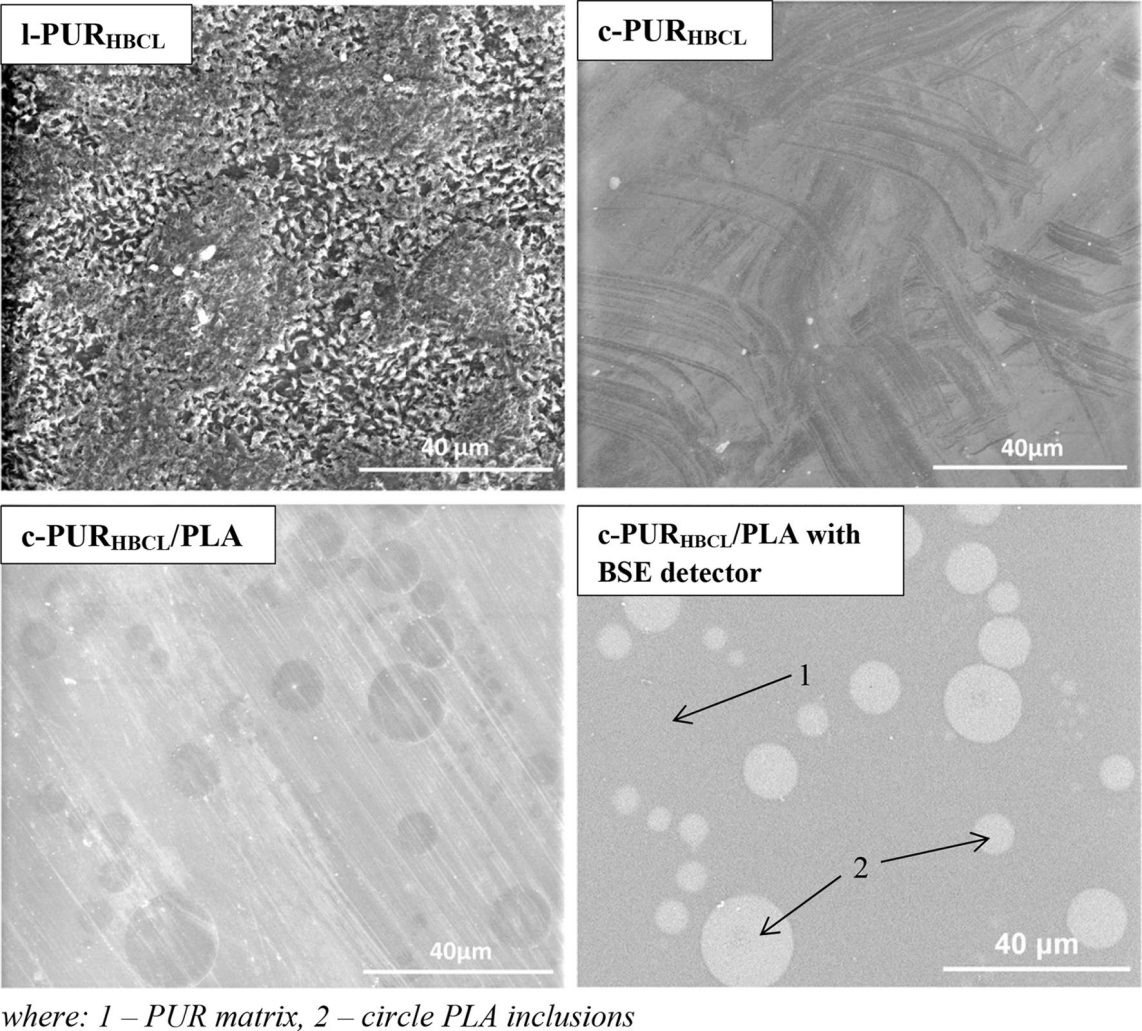
SEM micrographs of linear l-PUR_HBCL_, cross-linked c-PUR_HBCL_ and its blend, and image of c-PUR_HBCL_/PLA made with using of BSE detector. 1 means PUR matrix; 2 means circle PLA inclusions

Macroscopic evaluation of linear l-PUR_HBCL_ and cross-linked c-PUR_HBCL_ as well its blend with PLA (c-PUR_HBCL_/PLA) was also performed using scanning electron microscopy. The SEM images of samples are presented in (Fig. 5).

The results obtained from SEM analysis were similar to observation under optical microscopic and confirmed that the morphology of surface for linear and cross-linked PUR was different.

From Fig. 5, it was found that the linear PURs exhibited a regular characterized microstructure with grid patterns on the surface (Fig. 5, in Top/Left corner), while the cross-linked PURs display relatively smooth and compact surface (Fig. 5, in Top/Right corner). Pergal et al. (2014b) also observed that polyurethanes with lower crystallinity possessed surface smoother than those with high degree of crystallinity.

In addition, in SEM images of PUR/PLA blends, the circle inclusions with different diameters and un-uniformly distributed were visible (Fig. 5, in Bottom/Left corner). Due to PUR and PLA immiscibility and in case of low amount of PLA in blend, a droplet-like dispersion phase was created in PUR matrix (Liu et al. 2017). Clear phase boundary between these two polymers (PUR and PLA) was confirmed using BSE (Fig. 5, in Bottom/Right corner). This phase-separated morphology of blend disordered of PUR structure and changed the thermal properties (Table 2).

Energy-dispersive spectra (EDS) of cross-linked c-PUR_HBCL_ and its blend c-PUR_HBCL_/PLA were done for area of matrix (1) and circle inclusions (2) and well showed differences in chemistry of these regions (Fig. 5, in Bottom/Right corner). Mass concentrations, calculated for carbon and oxygen (Table 3), confirmed that round aggregates (bright circular spots) were consisted with PLA, whereas dark homogenous matrix was built with PUR.

**Table 3:** Carbon (C) and oxygen (O) concentrations in matrix (1) and circle inclusions (2) of c-PUR_HBCL_ and c-PUR_HBCL_/PLA calculated from energy-dispersive spectra (EDS)

Sample	C (wt%)	O (wt%)
c-PUR_HBCL_	73.7	26.3
c-PUR_HBCL_/PLA (1)	72.7	27.3
c-PUR_HBCL_/PLA (2)	61.1	38.9

## Conclusion

Two endothermic phase transitions (connected with melting of crystalline phase of soft and hard domains) were observed on DSC thermograms of PURs and their blends. Linear PURs were more crystalline than cross-linked (melting enthalpies of soft segments of l-PUR_HBCL_ and c-PUR_HBCL_ were 48.9 and 9.1 J/g, respectively). Blending of linear PUR with polylactide disturbed the crystalline structures formation (ΔH_1_ reduced to 26.8 J/g) opposite to cross-linked PURs for which PLA acted as plasticizers (the chain ordering was growing and ΔH_1_ increased to 18.6 J/g).

The linear PURs had a more granular surface, whereas surface of cross-linked PURs was smooth and flat. The presence of crystallites in linear l-PUR_HBCL_ bulk caused that the surface was formed at the film–air interface as the rock-like structure. Blending of l-PUR_HBCL_ with PLA caused that its crystallinity was decreased and surface was less rough, whereas l-PUR_HBTMG_ had become rougher and irregular. Surface of cross-linked c-PUR_CL_ (which is almost amorphous polymer) was the most irregular among all cross-linked PURs. In matrix of PUR blends, the circle inclusions of polylactide were visible.

Depending on the structure and the associated surface morphology, the obtained materials will be characterized by different physicochemical properties that could predispose them to medical or agricultural applications.
